# Clinical Profile of a Series of Left-Sided Prosthetic Valve Endocarditis: Revisiting Surgical Indications

**DOI:** 10.3390/diagnostics16030426

**Published:** 2026-02-01

**Authors:** Adrián Lozano Ibáñez, Javier López Díaz, María de Miguel Álava, Gonzalo Cabezón Villalba, Andrea Oña Orive, Daniel Gómez-Ramírez, Patricia Landín, Daniel Pérez-Camargo, Sofía Campillo, Itziar Gómez-Salvador, Carmen Sáez, Carmen Olmos, Isidre Vilacosta, J. Alberto San Román

**Affiliations:** 1 Servicio de Cardiología, Instituto de Ciencias del Corazón (ICICOR), Hospital Clínico Universitario de Valladolid, 47003 Valladolid, Spain; 2 Centro de Investigación Biomédica en Red de Enfermedades Cardiovasculares (CIBERCV), 28029 Madrid, Spain; 3 Instituto de Investigación Biosanitaria de Valladolid (IBioVALL), 47010 Valladolid, Spain; 4 Instituto Cardiovascular, Hospital Clínico San Carlos, Instituto de Investigación Sanitaria del Hospital Clínico San Carlos (IdISSC), 28040 Madrid, Spain; 5 Sección de Enfermedades Infecciosas, Servicio de Medicina Interna, Hospital Universitario de la Princesa, Instituto de Investigación Sanitaria del H.U. Princesa (IIS-IP), 28006 Madrid, Spain; 6 Facultad de Medicina, Salud y Deportes, Universidad Europea de Madrid, 28670 Madrid, Spain; 7 Department of Medicine, Universidad Complutense de Madrid, 28040 Madrid, Spain

**Keywords:** prosthetic endocarditis, surgical indications, clinical outcomes

## Abstract

**Background/Objectives:** Prosthetic valve endocarditis (PVE) carries a high morbidity and mortality. Surgery is classically indicated for heart failure, uncontrolled infection, or prevention of embolic events; however, evidence supporting surgery for “non-classical” indications—such as *Staphylococcus aureus* infection, non-HACEK Gram-negative bacteria, or early PVE—remains limited. This study aimed to update the clinical and prognostic profile of PVE and assess the impact of surgery, particularly in patients with non-classical surgical indications. **Methods**: We prospectively included all definite left-sided PVE cases diagnosed between 2000 and 2024 at three tertiary centers. Clinical, microbiological, echocardiographic, and prognostic data were analyzed and compared. Predictors of in-hospital mortality were identified using multivariable logistic regression, and Kaplan–Meier curves were used to compare 1-year survival according to surgical indications. **Results**: Among 589 patients with left-sided PVE, 61% underwent surgery, and in-hospital mortality was 31%. Independent mortality predictors were chronic obstructive pulmonary disease, pulmonary hypertension, periannular complications, *S. aureus* infection, and poor clinical condition at admission. Non-classical surgical indications were present in 38% of patients, although only 28 (5%) of them had no other surgical indication. These patients exhibited lower mortality (14%) and no survival benefit from surgery (10% vs. 17%; *p* 0.999). **Conclusions**: PVE remains associated with high mortality. Surgery improves survival in patients with classical surgical indications but not in patients with non-classical indications, supporting individualized surgical decisions by a multidisciplinary endocarditis team.

## 1. Introduction

Prosthetic valve endocarditis (PVE) is one of the most serious complications following valve replacement surgery due to its high morbidity and mortality, leading to aggressive treatment recommendations. Its prevalence has risen in the past decades from 5% to almost 20%, and its incidence in patients with surgical valves ranges from 0.5 to 1% per patient-year [1]. Mortality in PVE is significantly higher than in native valve endocarditis (NVE) [2], with recent data from a multicenter prospective registry reporting a 37.4% first year mortality in PVE compared with 31.6% in NVE [3].

The three classical indications for surgery in patients with left-sided infective endocarditis (IE) are heart failure, uncontrolled infection, and prevention of embolic events [4]. The 2015 European guidelines added PVE caused by staphylococci or non-HACEK Gram-negative bacteria (GNB) [5]. However, these non-classical recommendations are primarily based on older observational studies, which reported worse outcomes in patients who did not undergo surgery [6,7]. Notably, in 58.2% of these cases, the reason for not performing surgery was unknown, likely reflecting an older and more comorbid patient population [8]. The most recent 2023 guidelines [9] introduced early PVE—defined as occurring within six months of valve surgery—as a new class I surgical indication, also based on low-quality evidence.

The aim of our investigation is to update the epidemiological, clinical, echocardiographic, microbiological and prognostic profile of patients with PVE. In addition, we evaluated the impact of surgery when indicated for reasons other than heart failure, uncontrolled infection or prevention of embolic events.

## 2. Materials and Methods

### 2.1. Population

We prospectively identified all cases of definite left-sided prosthetic valve endocarditis diagnosed at three tertiary referral centers between 2000 and 2024, applying the diagnostic criteria in force at each time period (modified Duke criteria from 2005 to 2014, ESC 2015 criteria between 2015 and 2022, and the ESC 2023 revised diagnostic criteria from 2023 onwards) [5,9]. All patients with involvement of left-sided prosthetic valves were eligible for inclusion.

Transthoracic and transesophageal echocardiography were systematically performed in all cases. Image acquisition, measurements, and diagnostic assessment were conducted in accordance with the European echocardiographic recommendations [10].

Surgical intervention was primarily indicated in the presence of refractory heart failure, septic shock, ongoing infection despite appropriate antimicrobial therapy, fungal endocarditis, or recurrent systemic embolization. Decisions regarding surgical management were consistently made by a dedicated infective endocarditis Heart Team, comprising cardiologists, cardiac surgeons, and infectious disease specialists.

### 2.2. Variables Studied

We assessed up to 85 epidemiological, clinical, laboratory, microbiological, and echocardiographic variables collected at admission and during hospitalization, as well as treatment and outcome data. Missing data were below 10% for all variables.

### 2.3. Definition of Terms

Definition of early PVE has varied across guidelines and over time. In this study, early PVE was defined as PVE occurring within the first 6 months after valve surgery, in line with the most recent guidelines. Referred cases were defined as patients transferred from other healthcare centers where they were initially admitted. Heart failure was diagnosed by a clinical cardiologist following ESC guideline criteria [11], and was considered a surgical indication when resulting from valve dysfunction. Acute symptom onset was established using a 15-day cutoff between symptom onset and hospital admission.

Nosocomial infective endocarditis was defined as IE developing ≥72 h after hospital admission or associated with an in-hospital procedure performed within the previous 8 weeks [9]. Renal failure was defined as serum creatinine > 2 mg/dL in patients with previously normal renal function or a >50% increase in creatinine in those with chronic kidney disease [12]. Chronic anemia was defined as hemoglobin levels < 13 g/dL in men and <12 g/dL in women persisting for at least one year [13]. Persistent infection was defined as ongoing fever and positive blood cultures after 7 days of appropriate antibiotic therapy, once alternative causes of fever and perivalvular complications had been excluded [14]. Uncontrolled infection as an indication for surgery was defined as persistent infection, septic shock, locally uncontrolled infection (including perivalvular complications), or infection caused by resistant microorganisms, including multidrug-resistant bacteria and fungi, in accordance with the latest ESC guidelines. Systemic embolic events were diagnosed using imaging modalities such as computed tomography or ultrasonography and were considered a surgical indication after one or more embolic events attributable to infective endocarditis despite appropriate antibiotic therapy [9]. Decisions to withhold indicated surgery because of prohibitive risk were made by consensus of the Heart Team.

Non-classical surgical indications were defined as surgical decisions made for reasons other than the traditional indications of heart failure, uncontrolled infection, or prevention of embolic events, specifically PVE caused by *Staphylococcus aureus*, non-HACEK Gram-negative bacteria, or early PVE.

### 2.4. Statistical Analysis

Frequencies and percentages were reported for categorical variables. The chi-square test (or Fisher’s exact test when expected frequencies were less than 5) was used to compare groups. For continuous variables, the Kolmogorov–Smirnov test and Q-Q plots were used to verify normal distribution; these variables were reported as mean ± standard deviation or median [interquartile range, IQR] depending on their distribution. To compare continuous variables between groups, Student’s *t*-test or Mann–Whitney U test was used.

To analyze the prognostic factors of in-hospital mortality in patients with prosthetic valve endocarditis (PVE), a backward stepwise logistic regression model was used. Age, acute onset, COPD, pulmonary hypertension, periannular complication, early PVE, viridans streptococci, *Staphylococcus aureus*, heart failure at admission, septic shock at admission, renal failure at admission, and surgery were included in the model. To avoid overfitting, the variable-to-event ratio was controlled for all adjusted models. Adjusted odds ratios (OR) and their 95% confidence intervals (95% CI) were calculated. Non-collinearity was checked, and the model’s performance was evaluated using the area under the receiver operating characteristic curve (AUC-ROC) for discrimination, and the Hosmer–Lemeshow test for calibration.

To further address potential selection bias regarding surgical indications, an Inverse Probability of Treatment Weighting (IPTW) analysis was performed considering the following variables: age, referred, acute onset, cancer, anemia, COPD, viridans streptococci, staphylococcus aureus, periannular complication, vegetation, severe regurgitation, heart failure, acute kidney injury, septic shock and stroke. All variables included in the propensity score model were baseline characteristics assessed at admission, prior to the surgical decision. Propensity scores were estimated using a multivariable logistic regression model. Stabilized weights were calculated to minimize the influence of outliers, and weight trimming at the 1st and 99th percentiles was applied to ensure model stability. Covariate balance was assessed using standardized mean differences (SMD), where an SMD < 0.1 indicated adequate balance.

Survival at 1-year was estimated using the Kaplan–Meier method. For the IPTW-adjusted analysis, weighted Kaplan–Meier curves were generated. Statistical significance for survival comparisons was determined using the log-rank test for unadjusted data and a weighted Cox proportional hazards model with robust sandwich variance estimators for IPTW-adjusted data.

Statistical analyses were conducted using R software (version 4.4.2; R Foundation for Statistical Computing, Vienna, Austria) and Python (version 3.12; Python Software Foundation, Wilmington, DE, USA). Specifically, IPTW modeling, robust variance estimations, and weighted survival visualizations were implemented using the statsmodels, lifelines, and pyreadstat libraries in Python. All hypothesis tests were two-sided with a significance level of 5%.

## 3. Results

Among 1761 patients with definitive IE included in our database, 1482 were left-sided, and 589 (40%) were left-sided PVE: 304 patients with aortic prostheses (52%), 230 with mitral prostheses (39%) and 55 patients with both prosthetic valves (9%). Mechanical valves were present in 347 patients (59%) and biological valves in 237 patients (40%). Up to five patients had both mechanical and biological valves (1%).

### 3.1. Characteristics of Patients with PVE

Table 1 summarizes the main epidemiological, clinical, microbiologic, echocardiographic and prognostic characteristics of patients with PVE, and it compares operated and non-operated patients.

A total of 589 patients were included, of whom 359 (61%) underwent surgery. The mean age was 68.6 ± 12 years, and 62.5% were male. Cardiovascular risk factors and comorbidities were common, including diabetes (26%), chronic kidney impairment (18%), and COPD (10%), and 11% had a history of cancer. Microbiological diagnosis was available in most cases, with *Staphylococcus aureus* (17%), coagulase-negative staphylococci (26%), and enterococci (15%) as the most frequent pathogens; blood cultures were positive at admission in 76% of patients. At presentation, complications such as heart failure (40%), acute kidney injury (23%), and embolic events (17%) were frequent. Echocardiography showed vegetations in 78% of cases, periannular complications in 36%, and severe regurgitation in 27%. In-hospital mortality remained high (31% overall), with nearly half of deaths occurring in the surgical cohort.

Patients not undergoing surgery were older (72 ± 11 vs. 66 ± 12 y.o; *p* < 0.001) and had a higher burden of comorbidities than those who underwent surgery. *S. aureus* infection was more frequent among non-operated patients (21.4% vs. 13.4%; *p* 0.01), whereas fungal (1.3% vs. 5.3%; *p* 0.013) and anaerobic infections (1.3% vs. 6.7%; *p* 0.002) were more common in operated patients. Acute kidney injury (AKI) and septic shock at admission were more frequent in non-surgical patients (30.7% vs. 18.1%; *p* < 0.001, and 10.5% vs. 5.6%; *p* 0.026, respectively), while the frequency of heart failure, embolic events, and stroke did not differ between groups.

### 3.2. Surgical Indications

**Figure 1:**
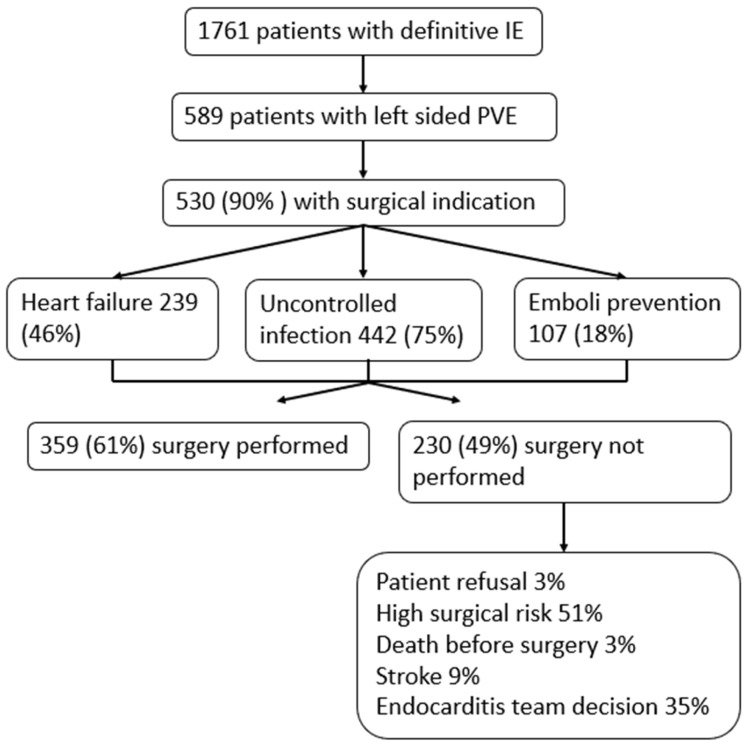
Flowchart showing patients with definite infective endocarditis (IE) and left-sided prosthetic valve endocarditis (PVE), surgical indications, and reasons for not undergoing surgery.

A flowchart of surgical indications can be found in Figure 1. Of the 589 patients with PVE, 530 (90%) had at least one surgical indication: 239 heart failure (46%), 442 uncontrolled infection (75%), 107 embolism prevention (18%) and 226 “non-classical indications” (44%). Many patients had more than one indication. Overall, 359 patients (61%) ultimately underwent surgery. The reasons for not undergoing surgery among the remaining patients were as follows: patient refusal (3%), high risk (51%), death prior to surgery (3%), stroke (9%) and Endocarditis Team decision (35%).

### 3.3. Mortality Among Patients with PVE

In-hospital mortality of patients with PVE in our cohort was 31%. Non-survivors were older (70.9 vs. 67.6 y.o, *p* < 0.001) and had higher prevalence of comorbidities, including diabetes (34% vs. 23%, *p* 0.004), COPD (16% vs. 8% *p* 0.001), and chronic kidney disease (27.6% vs. 12.9%, *p* < 0.001). From a microbiological perspective, viridans streptococci were more frequent among survivors (11% vs. 3%; *p* 0.001), whereas *S. aureus* was more common among non-survivors (11% vs. 29%; *p* < 0.001). Among operated patients, the highest mortality was observed in those with uncontrolled infection (28%), followed by heart failure (27%). Time delay between indication and surgery once it was indicated by the Endocarditis Team was similar in survivors and non-survivors (2 days [1–5] vs. 2 [0–5], *p* 0.247).

However, mortality in operated patients was similar to that of patients who did not undergo surgery despite having a formal surgical indication when this decision was taken by the multidisciplinary team (15% vs. 25% in operated patients, *p* 0.124).

In multivariable analysis (Table 2), COPD, pulmonary hypertension, periannular complications, *S. aureus* infection, and poor clinical condition at admission (heart failure, septic shock, and renal failure) were independently associated with in-hospital mortality. Viridans streptococci infection and surgery were associated with improved survival.

After inverse probability of treatment weighting (IPTW) adjustment, covariate balance between surgical and non-surgical patients markedly improved, with all standardized mean differences reduced to below 0.10. The association between surgery and lower in-hospital mortality persisted with a magnitude similar to that observed in unadjusted and multivariable analyses (Supplementary Table S1 and Supplementary Figure S1).

### 3.4. Patients with ‘’Non-Classical’’ Indications for Surgery

A total of 226 (38%) patients had a “non-classical surgical indication”. However, most of these patients had also a classical indication (88%): persistent infection was present in 187 (83%), heart failure in 80 (35%), and emboli prevention in 66 (29%). This left 28 patients with only non-classical surgical indications: early PVE in 18 patients (including 4 caused by *S. aureus* and 3 by non-HACEK Gram-negative bacteria), isolated *S. aureus* infection in 8 patients, and isolated non-HACEK Gram-negative bacterial infection in 2 patients. The characteristics of these patients are shown in Table 3.

The mean age was 66.8 years, and 43% were male. Comorbidities were common, including diabetes (36%) and CKD (18%). *S. aureus* (43%) and coagulase-negative staphylococci (21%) were the most frequent pathogens; blood cultures were positive in 96% of cases, and 36% of patients ultimately underwent surgery. Overall, in-hospital mortality of patients with non-classical surgical indication was 14%, and there were no differences in mortality between patients undergoing surgery (*n* = 10) or medical treatment (*n* = 18) (10% vs. 16.7%; *p* 0.999). Survivors were younger (65 vs. 77.5 y.o; *p* 0.045) and less frequently presented with heart failure at admission (17% vs. 75%; *p* 0.038). No other significant differences between survivors and non-survivors were observed in the variables analyzed.

A comparison between patients with and without classical surgical indications is shown in Table 3. Patients with non-classical surgical indications were more often of nosocomial origin (61% vs. 31%, *p* 0.001) and presented more frequently with acute onset (82% vs. 59%, *p* 0.021). As expected, *S. aureus* (43% vs. 17%, *p* 0.001) and GNB (18% vs. 5%; *p* 0.014) were more common in this group. Regarding echocardiographic features, patients with classical surgical indications had aortic involvement more frequently (64% vs. 32%; *p* 0.001), whereas mitral involvement was more common in patients with non-classical indications (71% vs. 46%; *p* 0.009). Periannular complications and severe regurgitation were also more frequent in patients with classical surgical indications. Vegetations tended to be more frequent in patients with non-classical indications (77.7% vs. 92.9%; *p* 0.058). However, in-hospital mortality was significantly lower among patients with non-classical indications (14% vs. 35%%, *p* 0.024).

**Figure 2:**
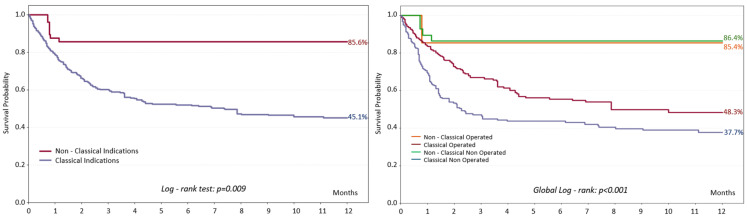
IPTW-adjusted one-year survival according to surgical indications and surgical treatment.

IPTW-adjusted Kaplan–Meier survival curves are shown in Figure 2. Patients with classical surgical indications had worse 1-year outcomes than those with non-classical indications. On the other hand, surgery improved prognosis in patients with classical surgical indication but not in those without classical surgical indication. Unadjusted curves can be found in Supplementary Figure S2.

## 4. Discussion

PVE remains one of the most challenging forms of IE due to its diagnostic complexity, microbiological heterogeneity, and high morbidity and mortality. There is a need to update the evidence base supporting surgical indications in PVE using contemporary data that reflect advances in surgical techniques and the increasing frailty of today’s patient populations.

In this large, multicenter cohort of 589 patients with left-sided PVE, we provide updated epidemiological, microbiological and prognostic insights. The main findings in our study were as follows: (1) PVE has high overall mortality and surgery improves prognosis; (2) most patients with non-classical indications for surgery nonetheless exhibit classical surgical indications, and both groups display a similar clinical profile; (3) patients with isolated “non-classical indications” have better prognosis than patients with classical indications and may not benefit from surgery in all cases.

### 4.1. Prognostic Factors and Determinants of Mortality

Our cohort confirms that the current epidemiological profile of patients with PVE is characterized by advanced age and frequent comorbidities, consistent with recent studies [3]. Compared with NVE, these patients are older and more fragile, which makes surgical decision making more challenging. From a microbiological standpoint, *Staphylococcus aureus* and coagulase-negative staphylococci were the predominant pathogens, probably due to their strong affinity for prosthetic material and biofilm-forming ability [15,16]. Our results reinforce previous observations that PVE remains a distinct clinical entity with a significant in-hospital mortality, in line with the 30–40% mortality reported in other large registries [3,8,17].

Predictors of mortality in our series were also consistent with the literature. *S. aureus* emerged as an independent predictor of mortality [18,19], whereas viridans streptococci were associated with better prognosis, possibly reflecting a less virulent course. Persistent positive blood cultures after 48 h of antibiotic treatment also identified patients at higher risk [20]. Other factors previously associated with poor prognosis—heart failure, septic shock, acute renal failure, pulmonary hypertension, and periannular complication—were also present in our cohort [19,21], but only periannular complication was linked to a higher referral to surgery.

### 4.2. Evidence of Non-Classical Surgical Indications

Surgical indication rates in PVE are consistently high, reaching 75% in previous series [3]. In our cohort they were even higher, partially reflecting the high proportion of referred patients, who likely represented the most severe cases.

Current guidelines recommend surgery with a class I indication in early PVE [9], yet the supporting evidence remains limited. Classical series reported higher mortality in early compared with late PVE, mainly driven by a higher burden of severe complications, but outcomes were not clearly improved by systematic surgery. A study in patients with PVE reported a mortality twice as high in patients with early PVE compared to late PVE (47.2% vs. 23%, *p* 0.003), and, although the study did not directly compare medical versus surgical treatment, the authors suggested a benefit of early surgical approach in patients with early PVE due to its association with increased mortality, largely attributed to a higher frequency of severe complications such as heart failure, intracardiac abscesses, persistent bacteremia, and stroke [17].

Similarly, Castillo et al. [22] reported higher in-hospital mortality in early PVE compared to late PVE (31% vs. 9%), although 4-year event-free survival did not differ significantly between groups. The proportion of patients undergoing surgery was similar in both groups, and the worse prognosis observed in early PVE was again linked to a higher incidence of heart failure. Based on these findings, the authors recommend early surgery only in patients with complicated early PVE [22]. Another very recent series of 302 patients with PVE found no differences in 1-year mortality between early (≤6 months) and late PVE cases (21% vs. 32%, *p* 0.126) [23]. This suggests that worse prognosis in early PVE is largely mediated by complications rather than by timing itself.

Regarding IE caused by *S. aureus*, Chirouze et al. [24] reported a cohort of 61 patients with *S. aureus* PVE, showing a high mortality rate (47.5%). Early surgery did not improve survival except in patients with “cardiac complications,” including heart failure or intracardiac abscess [24]. Other studies failed to demonstrate a mortality benefit of surgery in *S. aureus* PVE when classical indications were absent [18], suggesting that microbiology alone may be insufficient to justify surgical intervention.

Our analysis of patients with non-classical surgical indications suggests that this group has an outcome not as poor as initially expected, irrespective of whether surgery was performed, which is maintained at 1-year follow-up and also after IPTW adjustment. Therefore, although the sample size precludes definitive conclusions, these findings question the routine surgical indication in all patients without classical indications and reinforce the importance of the endocarditis team.

### 4.3. Selection Bias

Moreover, the consistently lower mortality observed in surgically treated patients across studies is probably influenced by selection bias, as frailer and older patients are often excluded from surgery despite having an indication. Interestingly, in our cohort, mortality was similar in operated and non-operated PVE patients despite having surgical indications when the decision was taken by the Endocarditis Team, highlighting the importance of individualized risk assessment as supported by prior studies, including the EURO-ENDO registry [8].

A study from Lalani et al. [25] demonstrated that early valve replacement was not associated with lower mortality compared with medical therapy in PVE after appropriate adjustment for differences in clinical characteristics and survival bias using surgery as a time-dependent covariate. Of note, subgroup analyses showed that the potential benefit of surgery was largely confined to patients with the highest surgical propensity, characterized by severe valvular dysfunction, heart failure, new valvular regurgitation, and perivalvular or prosthetic complications, whereas no clear benefit was observed in patients undergoing surgery solely for the presence of vegetations or embolic risk prevention [25].

In our IPTW analysis based on the probability of undergoing surgery, this adjustment did not change the association between surgery and lower mortality in the overall cohort. These findings suggest that the observed benefit is not solely explained by baseline differences between surgical and non-surgical patients. Nevertheless, residual confounding and survival bias inherent to observational studies cannot be completely excluded.

### 4.4. Limitations

This study has several limitations. First, bias cannot be excluded as it is an observational analysis of a prospective cohort. Second, the number of patients with only ‘’non-classical surgical indications’’ was small, which limits the statistical power and conclusions regarding this subgroup. Third, given the long inclusion period, changes in diagnostic criteria, imaging modalities, and surgical techniques may have influenced outcomes. Finally, these findings may not be generalizable to non-referral centers or to institutions lacking surgical facilities or a dedicated endocarditis team. Fourth, IPTW analysis included several baseline variables related to frailty, although no score was used.

## 5. Conclusions

PVE remains a severe condition associated with high morbidity and mortality. Our findings support a selective approach to surgical decision making, emphasizing individualized risk–benefit evaluation within a dedicated endocarditis multidisciplinary team. Future guidelines could aim to adapt surgical recommendations to modern data that integrate clinical presentation, surgical risk, and complications.

## Figures and Tables

**Table 1:** Characteristics of patients with PVE and comparison between operated and non-operated patients.

	Total (589)	No Surgery (230)	Surgery (359)	*p* Value
Epidemiologic variables				
Age, years	68.6 ± 12	72 ± 11	66 ± 12	<0.001
Male	62.5% (368)	61.3% (141)	63.2 (227)	0.638
Referred	43.8% (258)	34.4% (77)	51.4% (181)	<0.001
Acute onset	60.2% (353)	66.4% (152)	56.3% (201)	0.015
Diabetes	26.3% (155)	30.9% (71)	23.4% (84)	0.045
Cancer	10.5% (62)	14.3% (33)	8.1% (29)	0.016
Anemia	23.6% (139)	29.6% (68)	19.8% (71)	0.007
COPD	10.4% (61)	13.9% (32)	8.1% (29)	0.024
Chronic kidney injury	17.5% (103)	22.6% (52)	14.2% (51)	0.009
Atrial fibrillation	16.8% (95)	18.4% (40)	15.9% (55)	0.425
Microbiological variables				
*Streptococcus gallolyticus*	3.9% (23)	3.5% (8)	4.2% (15)	0.676
Viridans streptococci	8.8% (52)	11.4% (26)	7.2% (26)	0.087
Enterococci	15.1% (89)	21.4% (49)	11.1% (40)	0.001
Other streptococci	2.6% (15)	2.6% (6)	2.5% (9)	0.932
*Staphylococcus aureus*	16.5% (97)	21.4% (49)	13.4% (48)	0.011
Coagulase-negative staphylococci	26.2% (154)	20.1% (46)	30.1% (108)	0.007
Gram-negative bacilli	4.9% (29)	4.8% (11)	5% (18)	0.909
Fungi	3.7% (22)	1.3% (3)	5.3% (19)	0.013
HACEK	0.3% (2)	0.4% (1)	0.3% (1)	0.999
Anaerobic bacteria	4.6% (27)	1.3% (3)	6.7% (24)	0.002
Negative cultures	14.6% (86)	13.5% (31)	15.3% (55)	0.551
Positive cultures at admission	76.2% (413)	82.5% (184)	71.8% (229)	0.004
Positive cultures after 48 h	30.8% (117)	34% (52)	28.6% (65)	0.268
Clinical variables at admission				
Emboli at admission	16.9% (99)	18.4% (42)	15.9% (57)	0.422
Heart failure at admission	39.9% (234)	38.6% (88)	40.7% (146)	0.617
AKI at admission	23% (135)	30.7% (70)	18.1% (65)	<0.001
Stroke at admission	11.1% (65)	11.4% (26)	10.9% (39)	0.839
Septic shock at admission	7.5% (44)	10.5% (24)	5.6% (20)	0.026
Pulmonary hypertension	30.6% (175)	29% (64)	31.6% (111)	0.501
Early PVE	22.2% (131)	18.7% (43)	24.5% (88)	0.098
Echocardiographic variables				
Aortic prosthesis	61% (359)	56.1% (129)	64.1% (230)	0.053
Mitral prosthesis	48.4% (285)	49.6% (114)	47.6% (171)	0.647
Mechanical prothesis	59.8% (352)	55.7% (128)	62.4% (224)	0.103
Biological prosthesis	41.1% (242)	44.3% (102)	39% (140)	0.198
Vegetation	78.3% (451)	83.9% (187)	74.8% (264)	0.01
Periannular complication	35.9% (207)	23.3% (52)	43.9% (155)	<0.001
Severe regurgitation	26.6% (153)	13% (29)	35.1% (124)	<0.001
Evolutive variables				
Septic shock	15.8% (93)	22.4% (51)	11.7% (42)	0.001
Emboli	26.4% (155)	28.5% (65)	25.1% (90)	0.357
Heart failure	49.2% (289)	50.4% (115)	48.5% (174)	0.642
Acute kidney injury	39% (229)	49.6% (113)	32.3% (116)	<0.001
Stroke	16.2% (95)	18.4% (42)	14.8% (53)	0.241
Mortality				
In-hospital mortality	31.4% (185)	41.3% (95)	25.1% (90)	< 0.001

Data are expressed as mean ± standard deviation for continuous variables and as percentages (absolute number) for categorical variables. COPD: chronic obstructive pulmonary disease; HACEK: Haemophilus, Aggregatibacter, Cardiobacterium, Eikenella, Kingella; and PVE: prosthetic valve endocarditis.

**Table 2:** Mortality predictors among patients with PVE.

	Univariate		Multivariate	
	OR (95%CI)	*p* Value	OR (95%CI)	*p* Value
Age	1.03 (1.09–1.04)	0.002		
Diabetes mellitus	1.75 (1.19–2.57)	0.004		
COPD	2.34 (1.37–4.01)	0.002	2.22 (1.21–4.09)	0.010
Pulmonary hypertension	1.80 (1.24–2.62)	0.002	1.75 (1.12–2.74)	0.014
Periannular complications	1.65 (1.15–2.37)	0.007	2.16 (1.41–3.32)	<0.001
Early PVE	0.68 (0.44–1.05)	0.083		
Viridans streptococci	0.26 (0.11–0.63)	0.003	0.36 (0.14–0.92)	0.033
*Staphylococcus aureus*	3.31 (2.12–5.18)	<0.001	2.94 (1.75–4.92)	<0.001
Heart failure at admission	2.20 (1.55–3.14)	<0.001	1.82 (1.18–2.74)	0.014
Septic shock at admission	4.30 (2.27–8.17)	<0.001	2.31 (1.09–4.90)	0.029
Renal failure at admission	3.16 (2.12–4.70)	<0.001	2.22 (1.40–3.52)	0.001
Surgery	0.48 (0.33–0.68)	<0.001	0.45 (0.30–0.70)	<0.001

Odds ratios (OR) are presented with 95% confidence intervals (CIs). COPD: chronic obstructive pulmonary disease; and PVE: prosthetic valve endocarditis.

**Table 3:** Comparison of patients with classical and non-classical surgical indications.

	Total (530)	Classical Indications (502)	Only Non-Classical Indications (28)	*p* Value
Epidemiological variables				
Age, years	68 ± 12	68.5 ± 11.9	66.8 ± 11.6	0.283
Male	62.5% (331)	63.5% (319)	42.9% (12)	0.028
Referred	46.6% (242)	47.4% (233)	33.3% (9)	0.155
Nosocomial	32.8% (174)	31.3% (157)	60.7% (17)	0.001
Acute onset	60.2% (318)	59.1% (296)	81.5% (22)	0.021
Diabetes mellitus	27.0% (143)	26.5% (133)	35.7% (10)	0.285
COPD	10.4% (55)	10.4% (52)	10.7% (3)	0.999
Cancer	10.4% (55)	10.8% (54)	3.6% (1)	0.343
Anemia	23.6% (125)	24.0% (120)	17.9% (5)	0.460
Chronic kidney injury	17.0% (90)	16.9% (85)	17.9% (5)	0.801
Atrial fibrillation	16.7% (86)	16.4% (80)	22.2% (6)	0.427
Microbiological variables				
*Streptococcus gallolyticus*	4.0% (21)	4.2% (21)	0.0% (0)	0.619
Viridans streptococci	7.4% (39)	7.0% (35)	14.3% (4)	0.143
Enterococci	14.6% (77)	15.0% (75)	7.1% (2)	0.406
Other streptococci	2.3% (12)	2.2% (11)	3.6% (1)	0.483
*Staphylococcus aureus*	18.3% (97)	17.0% (85)	42.9% (12)	0.001
Coagulase-negative staphylococci	26.5% (140)	26.7% (134)	21.4% (6)	0.535
Gram-negative bacilli	5.5% (29)	4.8% (24)	17.9% (5)	0.014
Fungi	4.2% (22)	4.2% (21)	3.6% (1)	0.999
HACEK	0.4% (2)	0.4% (2)	0.0% (0)	0.999
Anaerobic bacteria	4.5% (24)	4.8% (24)	0.0% (0)	0.630
Negative cultures	14.4% (76)	14.8% (74)	7.1% (2)	0.405
Positive cultures at admission	76.0% (373)	74.8% (347)	96.3% (26)	0.011
Positive cultures after 48 h	34.1% (117)	36.0% (117)	0.0% (0)	0.002
Clinical variables at admission				
Emboli at admission	17.7% (94)	17.7% (89)	17.9% (5)	0.999
Heart failure at admission	41.7% (221)	42.6% (214)	25.0% (7)	0.066
AKI at admission	23.2% (123)	23.7% (119)	14.3% (4)	0.251
Stroke at admission	11.7% (62)	12.0% (60)	7.1% (2)	0.760
Septic shock at admission	8.3% (44)	8.8% (44)	0.0% (0)	0.155
Pulmonary hypertension	31.7% (165)	32.5% (160)	17.9% (5)	0.106
Early PVE	24.7% (131)	22.5% (113)	64.3% (18)	<0.001
Echocardiographic variables				
Mitral prosthesis	47.4% (251)	46.0% (231)	71.4% (20)	0.009
Aortic prosthesis	62.6% (332)	64.3% (323)	32.1% (9)	0.001
Mechanical prosthesis	60.0% (318)	59.4% (298)	71.4% (20)	0.205
Biological prosthesis	40.9% (217)	41.6% (209)	28.6% (8)	0.171
Vegetation	78.5% (413)	77.7% (387)	92.9% (26)	0.058
Periannular complication	39.4% (207)	41.6% (207)	0.0% (0)	<0.001
Valve perforation	3.8% (20)	4.0% (20)	0.0% (0)	0.617
Severe regurgitation	28.7% (151)	30.3% (151)	0.0% (0)	0.001
Outcomes				
Surgery	63.2% (335)	64.7% (325)	35.7% (10)	0.002
In-hospital mortality	34.0% (180)	35.1% (176)	14.3% (4)	0.024

Data are presented as mean ± standard deviation for continuous variables and percentages (absolute numbers) for categorical variables. *p*-values refer to the comparison between classical (*n* = 502) and non-classical (*n* = 28) indication groups. COPD: chronic obstructive pulmonary disease; HACEK: Haemophilus, Aggregatibacter, Cardiobacterium, Eikenella, Kingella; PVE: prosthetic valve endocarditis; and AKI: acute kidney injury.

## Data Availability

The original contributions presented in this study are included in the article/Supplementary Materials. Further inquiries can be directed to the corresponding author.

## Supplementary Materials

The following supporting information can be downloaded at: https://www.mdpi.com/article/10.3390/diagnostics16030426/s1, Figure S1: (A) Standardized mean differences of covariates included in the IPTW weighting model. (B) Effect of Surgery on in-Hospital mortality; Figure S2: Unadjusted Kaplan–Meier curves for 1-year mortality in patients with classic and non-classical surgical indications; Table S1: Baseline characteristics and standardized mean differences before and after inverse probability of treatment weighting (IPTW) for surgical treatment.

## Abbreviations

The following abbreviations are used in this manuscript:

PVE	Prosthetic Valve Endocarditis
NVE	Native Valve Endocarditis
IE	Infective Endocarditis
HACEK	*Haemophilus*, *Aggregatibacter*, *Cardiobacterium, Eikenella*, and *Kingella* Group
GNB	Gram-Negative Bacteria
ESC	European Society of Cardiology
TTE	Transthoracic Echocardiography
TEE	Transesophageal Echocardiography
IQR	Interquartile Range
COPD	Chronic Obstructive Pulmonary Disease
OR	Odds Ratio
CI	Confidence Interval
ROC	Receiver Operating Characteristic
AKI	Acute Kidney Injury
CKD	Chronic Kidney Disease
