# Supplementary material for: Clinical Profile of a Series of Left-Sided Prosthetic Valve Endocarditis: Revisiting Surgical Indications
Source: Diagnostics (Basel). 2026 Feb 1;16(3):426. doi: 10.3390/diagnostics16030426 (PMC12897040; doi:10.3390/diagnostics16030426)

SUPPLEMENTARY MATERIALS

Table S1. Baseline characteristics and standardized mean differences before and after inverse probability of treatment weighting (IPTW) for surgical treatment

	No Surgery (Crude) n=230	Surgery (Crude) n=359	SMD Crude	No Surgery (IPTW) n=214	Surgery (IPTW) n=343	SMD Adjusted
**Epidemiologic variables**						
Age, years	72.2±11.0	66.3±12.0	0.519	69.1±12.8	68.3±11.3	0.061
Male	61.3% (141)	63.2% (227)	0.040	61.7%	60.1%	0.033
Referred	34.4% (77)	51.4% (181)	0.350	43.1%	45.6%	0.049
Acute onset	66.4% (152)	56.3% (201)	0.208	64.1%	60.0%	0.083
Diabetes mellitus	30.9% (71)	23.4% (84)	0.169	24.5%	22.4%	0.050
Cancer	14.3% (33)	8.1% (29)	0.199	10.7%	9.6%	0.037
Anaemia	29.6% (68)	19.8% (71)	0.227	24.8%	22.8%	0.048
COPD	13.9% (32)	8.1% (29)	0.187	11.8%	9.9%	0.062
Chronic kidney injury	22.6% (52)	14.2% (51)	0.218	15.5%	16.0%	0.012
Atrial fibrilation	18.4% (40)	15.9% (55)	0.069	18.2%	14.3%	0.105
**Microbiological variables**						
Streptococcus gallolyticus	3.5% (8)	4.2% (15)	0.036	3.5%	3.9%	0.024
Viridans streptococci	11.4% (26)	7.2% (26)	0.142	9.0%	8.6%	0.013
Enterococci	21.4% (49)	11.1% (40)	0.281	20.9%	12.5%	0.227
Other streptococci	2.6% (6)	2.5% (9)	0.007	1.9%	2.4%	0.036
Staphylococcus aureus	21.4% (49)	13.4% (48)	0.213	19.4%	16.5%	0.076
Coagulase negative staphylococci	20.1% (46)	30.1% (108)	0.232	19.8%	28.0%	0.194
Gram negative bacilli	4.8% (11)	5.0% (18)	0.010	4.5%	5.6%	0.051
Fungi	1.3% (3)	5.3% (19)	0.224	0.8%	5.4%	0.266
HACEK	0.4% (1)	0.3% (1)	0.026	1.3%	0.2%	0.128
Anaerobic bacteria	1.3% (3)	6.7% (24)	0.277	2.8%	5.7%	0.146
Negative cultures	13.5% (31)	15.3% (55)	0.051	17.5%	12.8%	0.131
Positive cultures at admission	82.5% (184)	71.8% (229)	0.258	77.9%	75.8%	0.050
Positive cultures after 48h	34.0% (52)	28.6% (65)	0.116	34.8%	30.8%	0.085
**Clinical variables at admission**						
Early PVE	18.7% (43)	24.5% (88)	0.142	27.0%	22.4%	0.107
Emboli at admission	18.4% (42)	15.9% (57)	0.068	15.9%	17.2%	0.035
Heart failure at admission	38.6% (88)	40.7% (146)	0.042	40.4%	42.1%	0.035
AKI at admission	30.7% (70)	18.1% (65)	0.296	27.2%	21.5%	0.131
Stroke at admission	11.4% (26)	10.9% (39)	0.017	9.7%	11.3%	0.053
Septic shock at admission	10.5% (24)	5.6% (20)	0.183	9.6%	7.3%	0.083
Pulmonary hypertension	29.0% (64)	31.6% (111)	0.058	28.2%	30.5%	0.051
**Echocardiographic variables**						
Aortic prosthesis	56.1% (129)	64.1% (230)	0.163	59.3%	63.0%	0.077
Mitral prosthesis	49.6% (114)	47.6% (171)	0.039	47.1%	48.9%	0.036
Mechanical prosthesis	55.7% (128)	62.4% (224)	0.137	59.3%	60.2%	0.019
Biological prosthesis	44.3% (102)	39.0% (140)	0.109	40.7%	41.0%	0.006
Vegetation	83.9% (187)	74.8% (264)	0.225	81.6%	77.7%	0.096
Periannular complication	23.3% (52)	43.9% (155)	0.447	34.4%	37.1%	0.057
Severe regurgitation	13.0% (29)	35.1% (124)	0.536	22.9%	27.5%	0.106
**Evolutive variables**						
Septic shock	22.4% (51)	11.7% (42)	0.287	18.3%	15.8%	0.067
Emboli	28.5% (65)	25.1% (90)	0.078	26.1%	27.1%	0.021
Heart failure	50.4% (115)	48.5% (174)	0.039	49.1%	49.4%	0.007
Acute kidney injury	49.6% (113)	32.3% (116)	0.356	41.3%	38.3%	0.062
Stroke	18.4% (42)	14.8% (53)	0.098	16.3%	15.0%	0.035
**Mortality**						
In-hospital mortality	41.3% (95)	25.1% (90)	0.350	39.8%	28.7%	0.237

Figure S1. (**A**) Standardized mean differences of covariates included in the IPTW weighting model. (**B**) Effect of Surgery on in-Hospital mortality

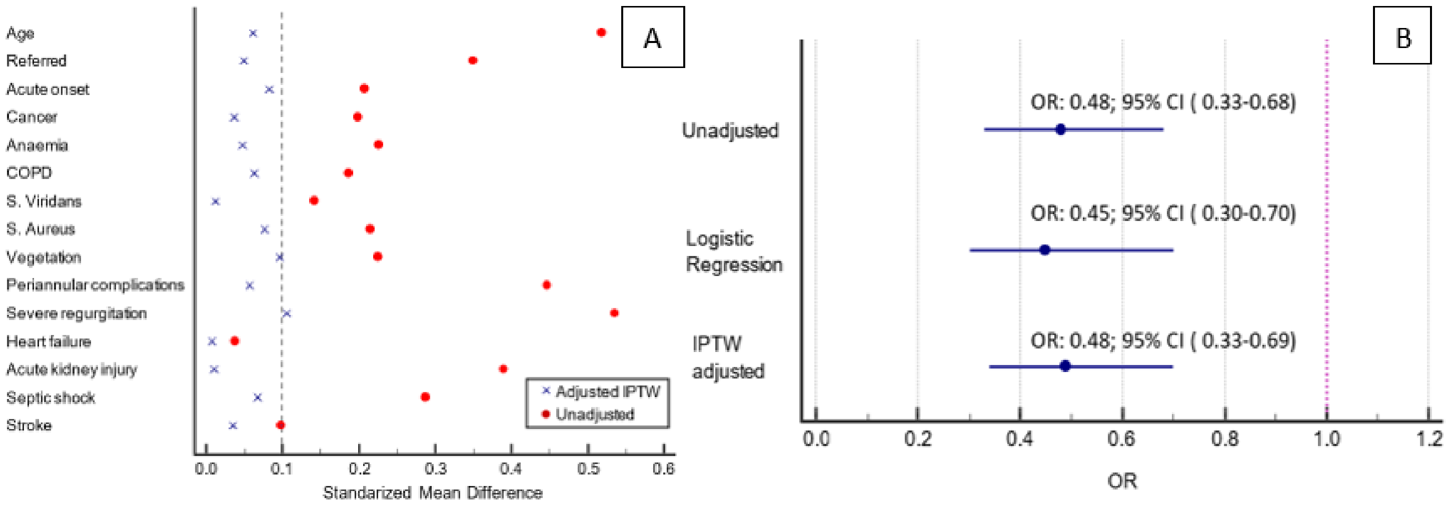

Figure S2. Unadjusted Kaplan–Meier curves for 1-year mortality in patients with classic and non – classical surgical indications.

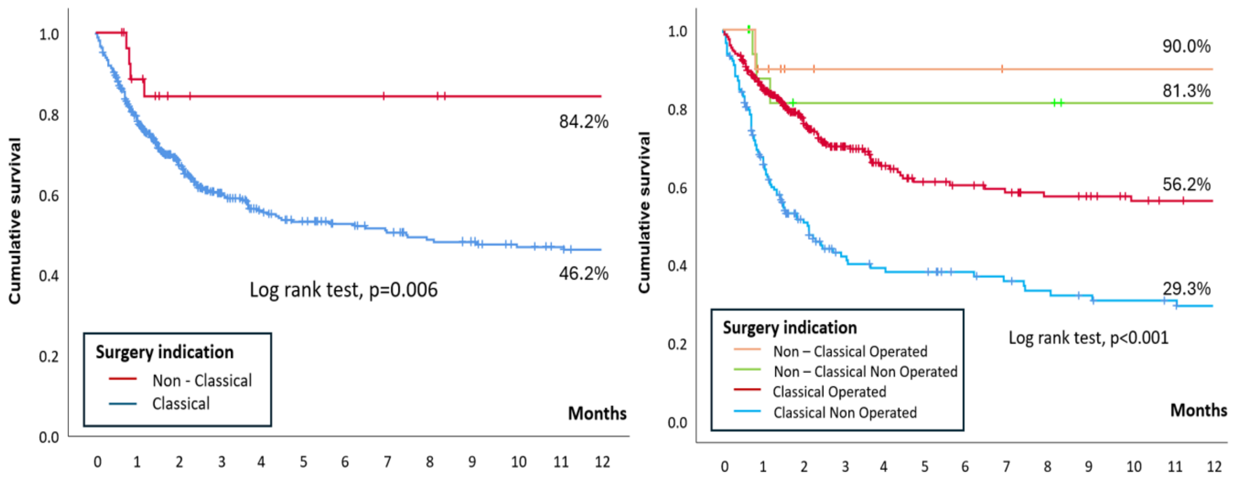

Supplement: Supplementary file 1 [file diagnostics-16-00426-s001.zip › diagnostics-4020915-supplementary.pdf]
